# Reducing relapses after acute malnutrition treatment: evidence from a simplified approach in emergency settings of Mali

**DOI:** 10.3389/fpubh.2026.1773585

**Published:** 2026-03-12

**Authors:** Pilar Charle-Cuéllar, Luis Javier Sánchez-Martínez, Sara Tulipani, Mamadou Saidou Diallo, Abdel Nasser Maïga, Amadou Tila Kebe, Fatimata Karabenta, Mahamadou N’tji Samake, Mohamed Ibrahim Mahmoud, André Briend, Antonio Vargas, Noemí López-Ejeda

**Affiliations:** 1 Nutrition and Health Department, Action Against Hunger, Madrid, Spain; 2 EPINUT Research Group (ref. 920325), Unit of Physical Anthropology, Department of Biodiversity, Ecology and Evolution, Faculty of Biological Sciences, Complutense University of Madrid, Madrid, Spain; 3 Nutrition and Health Department, Action Against Hunger, Bamako, Mali; 4 Nutrition Cluster, Bamako, Mali; 5 Ministry of Health, Bamako, Mali; 6 Department of Nutrition, Exercise and Sports, Faculty of Science, University of Copenhagen, Frederiksberg, Copenhagen, Denmark; 7 Center for Child Health Research, Faculty of Medicine and Medical Technology, Tampere University and Tampere University Hospital, Tampere, Finland

**Keywords:** children, community health worker, mid-upper arm circumference, ready-to-use therapeutic food, simplified protocol, wasting

## Abstract

**Background:**

Acute malnutrition remains a critical public health issue, particularly in emergency settings. While simplified treatment protocols show promise in improving access and cost-effectiveness, evidence on their impact on relapse rates remains limited. This study aimed to compare relapse incidence among children treated for severe acute malnutrition (SAM) using a simplified protocol versus the standard Community Management of Acute Malnutrition (CMAM) protocol in the emergency context of Gao, Mali.

**Methods:**

This is a non-randomized controlled trial conducted between December 2022 and December 2023, involving 506 children aged 6–59 months discharged as cured from SAM. The intervention group (*n* = 270) received treatment via a simplified protocol using Mid-upper arm circumference, (MUAC)-only criteria and fixed ready-to-use therapeutic (RUTF) dose, while the standard protocol group (*n* = 236) followed the standard CMAM protocol. Children were followed for up to eight months post-discharge. Relapse was defined as meeting anthropometric criteria for acute malnutrition during follow-up. Cox regression models were used to identify risk factors.

**Results:**

Relapse incidence was significantly lower in the simplified protocol group (5.6%) compared to the standard one (39.8%). The simplified protocol also had longer length of stay, used lower quantity of RUTF, and fewer comorbidities at discharge. We found similar results when analyzing the sub-sample with those children admitted with MUAC-only in the control group. Multivariate analysis identified treatment with the simplified protocol, older age at discharge, and greater MUAC gain as protective factors. Comorbidities during follow-up were the strongest predictor of relapse.

**Conclusion:**

Simplified protocols may reduce relapse rates after SAM treatment, even with reduced RUTF doses. Strengthening discharge procedures and post-discharge monitoring, particularly in emergency settings, is essential to sustain recovery and prevent relapse.

## 1 Introduction

Acute malnutrition is a serious public health issue affecting millions of children in the Global South. Despite being both preventable and treatable, more than 42.8 million (6.6%) children under the age of five suffer from it, including 12.2 million who experience severe acute malnutrition (SAM) (1), define as weight-for-height z-score (WHZ) < −3 and/or mid-upper arm circumference (MUAC) < 115 mm and/or mild bilateral pitting edema. This issue is not solely due to a lack of access to food or health services, but rather reflects a complex reality influenced by social and cultural norms that perpetuate gender inequality and poverty (2). Malnutrition is associated with 45% of all deaths among children aged 1–59 months, with the risk being particularly high among those suffering from SAM (3). According to the latest Standardized Monitoring and Assessment of Relief and Transitions (SMART) survey conducted in Mali in 2024 in the Gao region, the prevalence of moderate acute malnutrition (MAM) define as WHZ ≥ −3 to < −2 and/or MUAC ≥ 115 mm to < 125 mm and, no nutritional edema, is 15.7% [95% CI: 13.0–18.4], the prevalence of SAM is 3.6% [95% CI: 2.3–5.0] and the prevalence of global acute malnutrition (GAM) represents the sum of the prevalence of the 2 combined, is 19.3% [95% CI: 16.4–22.2] (4). These rates exceed the emergency thresholds established by the World Health Organization (WHO), which defines GAM above 15% as critical (5).

There is a close, bidirectional relationship between malnutrition and infectious diseases. Malnourished children are at increased risk of dying from infections due to immune system impairment. The integrity of skin, respiratory, and gastrointestinal mucosal barriers is compromised, further exacerbated by enteric dysfunction and alterations in the intestinal microbiota (6, 7). These children also exhibit elevated levels of pro-inflammatory cytokines, which disrupt the growth hormone IGF-1 axis, a process similarly observed in children with infectious diseases (8). Additionally, thymus atrophy and impaired T lymphocyte function reduce the body’s ability to fight infections (9). Consequently, malnourished children are more susceptible to infections, which in turn worsens their nutritional status. Infections often lead to reduced appetite and nutrient malabsorption, combined with increased metabolic demands, ultimately resulting in further nutritional deficits (10).

To address the substantial burden of morbidity and mortality associated with acute malnutrition, several initiatives have been implemented over recent decades. One of the most notable developments is the adoption of simplified approaches to the community-based management of acute malnutrition. These approaches aim to enhance the effectiveness and cost-effectiveness of modified treatment protocols while expanding coverage, particularly in hard-to-reach or crisis-affected areas (11). Within these approaches, it is proposed to simplify the protocol by using MUAC as the sole anthropometric criterion for admission, discharge, and follow-up, along with the provision of a fixed or reduced dose of ready-to-use therapeutic food (RUTF), independent of the child’s weight.

The main simplified protocols that demonstrated effectiveness, include ComPAS (Combined Protocol for Acute Malnutrition Study), implemented in stable settings across Global South countries (12–14), and in emergency contexts such as Mali and Niger (15–19), and OptiMA (Optimizing the Treatment of Acute Malnutrition), which also shown evidence of effectiveness in managing acute malnutrition (20–23). An additional intervention within simplified approaches is the treatment of acute malnutrition by community health workers (CHWs). This strategy seeks to bring care closer to population with a high risk of vulnerability by overcoming geographical, economic, and cultural barriers that limit access to health services. Evidence supporting this approach includes systematic review (24), an operational experience review (25), and multiple studies demonstrating early identification, treatment effectiveness, and coverage achieved by CHWs (26–36). These studies also highlight reductions in costs and improvements in cost-effectiveness (37–39).

In 2023, the WHO published the Guideline on the Prevention and Management of Wasting and Nutritional Oedema in Infants and Children Under 5 Years of Age. This guideline introduced major changes, including the integration of CHWs in treatment delivery, the inclusion of children under 6 months of age, and the recommendation to supplement children suffering from MAM. For the first time, it included prevention strategies aimed at reducing relapse and promoting sustained recovery after discharge. However, significant research gaps remain in identifying those children at higher risk of relapse (40).

The evidence on relapse is inconsistent: two recent systematic reviews report relapse rates to SAM ranging from 3 to 31%, depending on the context and relapse definition (41, 42). In countries such as Kenya, Burkina Faso, and Mali, relapse rates to SAM varied between 3, 3.7, and 1.7%, respectively (43–45). Most of these studies were conducted in stable contexts. Limited evidence exists on whether simplified protocols influence relapse risk.

The objective of this study is to estimate the incidence of relapse among children during the 8 months following recovery from uncomplicated SAM, comparing those treated with the standard Community Management of Acute Malnutrition (CMAM) protocol to those treated with a simplified protocol in the emergency context of Gao, northern Mali. In both groups, services were delivered through health facilities (HFs) and CHWs. The secondary objective is to identify variables that may help define a profile of children at greater risk of relapses.

## 2 Participants and methods

### 2.1 Study design and settings

This is a non-randomized controlled trial with two groups, comparing the incidence of relapse among children treated for uncomplicated SAM in the Gao region of northern Mali. The study area, Gao district, has a population of approximately 350,000 inhabitants. The population faces a complex humanitarian crisis, characterized by extreme weather conditions such as droughts and floods, compounded by significant economic and social challenges, as well as deteriorating security due to ongoing armed conflict over the past decade. In these rural areas, 57% of the population lives more than 5 km from a health facility, and 14% live more than 15 km away. According to the national primary health care policy, to address this lack of access to health services, CHWs must be located more than 5 km from a health facility and are expected to serve between 100 and 500 people within a 25 km radius (46).

Two communes were selected for the simplified protocol and two others for the standard one. The simplified protocol included 5 HFs and 5 CHWs and the standard one 6 HFs and 4 CHWs. The selection criteria were those HFs and CHWs located near the river, including HFs serving nomadic populations and those with the highest number of admissions according to the national information system. Children aged 6–59 months who were discharged as cured from uncomplicated SAM, having been treated with the simplified protocol, were included in the intervention group. This protocol used MUAC as the sole criterion for diagnosis, admission, discharge, and follow-up. Admission criteria were MUAC < 125 mm and/or mild bilateral pitting edema. Discharge criteria were MUAC ≥ 125 mm and absence of edema for two consecutive weeks. Children received a fixed daily dose of RUTF: 1000 kcal/day (2 sachets), except for children weighing less than 5 kg, who received 500 kcal/day (1 sachet); and children with MUAC between 115 and 125 mm, who received 1 sachet per day. There was no transition phase from 2 sachets to 1 sachet for children admitted with MUAC < 115 mm who MAM threshold of MUAC > 115 mm. Our study includes only those children with MUAC < 115 mm.

Children aged 6–59 months who were discharged as cured from SAM without complications, having been treated according to the standard protocol, were included in the standard protocol group. Admission criteria were WHZ < −3 and/or MUAC <115 mm and/or mild bilateral pitting edema. Discharge criteria were WHZ > −1.5 or MUAC ≥ 125 mm and absence of edema for two consecutive weeks. Children receive a daily dose of RUTF equivalent to 170 kcal/kg/day. In both groups, children also received systemic treatment with amoxicillin (50–100 mg/kg/day) twice a day for 5 days and one single dose of 500 mg of mebendazole at the first visit for deworming.

The sample size calculation was based on a dichotomous qualitative primary outcome (relapse: yes/no). A review and meta-analysis by O’Sullivan et al. (40) and Stobaugh et al. (40) indicated that the incidence of relapse in programs with standard protocols varies between 0 and 37%, depending on the country and definition. As there were no specific data available for Mali at the start of the study, the midpoint of this range (19%) was used as the reference value. Lelijveld et al. (40) found no significant difference in relapse incidence between children treated with the simplified protocol and those treated with the standard protocol (3% vs. 3%, *p* = 0.750). Consequently, the sample size was calculated under an equivalence assumption, in which both groups were expected to have a relapse incidence of approximately 19%, with an equivalence margin of 10%. Assuming a significant level (*α*) of 5% and a statistical power of 80%, the required sample size was 264 children per study group. Accounting for a 10% potential loss to follow-up, the final sample size was set at 290 children per group.

### 2.2 Implementation and ethical considerations

At the beginning of the study, an initial meeting was held with the health district to select the implementation sites and sign the collaboration agreement. For the inclusion of children, treatment providers obtained informed consent from all parents or caregivers in the local language. The Ethics Committee of the Institut National de Santé Publique (INSP), the reference agency of the Ministry of Health of the Government of Mali approved the study (Decision No. 35/2029/CE-EX-INRSP).

### 2.3 Procedures and data collection

Between December 2022 and December 2023, children aged 6–59 months diagnosed with uncomplicated SAM and discharged as cured from the selected treatment sites were included in the study. Variables collected at admission included: (1) Treatment-related variables, recorded retrospectively: age at admission; anthropometric data at admission and discharge (MUAC in the simplified protocol group, weight, height, and/or MUAC in the standard protocol group); presence of edema at admission; comorbidities (diarrhea, malaria, acute respiratory infection); length of stay (LoS) in treatment; total number of RUTF sachets received during treatment; time and distance to the treatment site; and caregivers’ ability to self-finance travel (assessed at the time of study enrolment); (2) Baseline variables, recorded at study enrolment: socio-demographic characteristics (village name, household size, presence of living parents, number of siblings under 5 years); household characteristics (ownership of property, number of rooms, building materials, presence of a separate kitchen, availability of a safe play space for children, access to electricity, ownership of land and livestock); maternal characteristics (years of schooling, age at pregnancy, number of prenatal consultations); family income; and household food security, measured using the Household Food Insecurity Access Scale (HFIAS), the most widely used indicator in studies on nutritional relapse to date (47); (3) Follow-up variables for relapse included anthropometric data (MUAC in the simplified protocol group, weight, height, and/or MUAC in the standard protocol group, define is the next section), presence of edema, and comorbidities (diarrhea, malaria, acute respiratory infection; Supplementary Table S1). Study supervisors collected treatment data directly from patient record books at the treatment sites using tablets, and socioeconomic data through direct interviews with caregivers at the health facilities or via CHWs, using the Kobo Toolbox app (48). The study was initially designed to conduct monthly follow-up visits for six consecutive months after being discharged from treatment. However, due to increasing security concerns, some HFs and CHWs were inaccessible during certain periods, preventing supervisors from carrying out scheduled visits. To mitigate the impact of this contextual limitation, several adjustments were made to the protocol. Ultimately, the research team was able to follow up with each child between one and four times, at varying intervals, with a minimum of two and a maximum of 8 months after discharge.

### 2.4 Outcomes

The primary outcome was relapse, defined as a child who had been discharged as cured from SAM treatment and who met the anthropometric criteria for acute malnutrition at any point during the follow-up period. In the simplified protocol group, relapse was defined as MUAC < 125 mm and/or the presence of nutritional edema. In the standard protocol group, relapse was defined as WHZ < −2 and/or MUAC < 125 mm and/or the presence of nutritional edema. Two types of relapses were recorded. Relapses to SAM defined when the child’s anthropometry meets the criteria for SAM (MUAC < 115 mm and/or WHZ < − 2 and/or presence of edema), during follow-up visits, and relapses to MAM defined when a child’s anthropometry meets the criteria for MAM (MUAC ≥ 115 mm to < 125 mm and/or WHZ ≥ −3 to < −2 z-score), during a follow-up visit.

Secondary outcomes of interest included LoS, the incidence of morbidities at any time during the post-discharge follow-up period, and other potential risk factors. Other co-variates as socio-economic variables were also analyzed (Supplementary Figure S1).

### 2.5 Statistical analysis

All analyses were performed using R (version 4.3.1) (49). Given the study design, in which each treatment site was assigned to one study group, a two-level hierarchical analysis was conducted to account for the data structure, including 20 sites with unbalanced sample sizes. To compare variables between study groups, mixed-effects models with random intercepts at the treatment site level were employed. Depending on the outcome variable, the following approaches were used: linear mixed-effects models with Satterthwaite’s correction for degrees of freedom for continuous outcomes, using the lmer() function from the lmerTest package; generalized linear mixed-effects models for binary outcomes, using the glmer() function from the lme4 package; and cumulative link mixed models for ordinal outcomes, using the clmm() function from the ordinal package. For statistically significant results (*p* < 0.05), the fitted models were compared to their corresponding null models (excluding the treatment variable) to isolate the effect of clustering, including assessment of the intraclass correlation coefficient.

To assess the impact of socioeconomic and treatment-related variables on relapse risk, Cox regression models were used, accounting for the clustered study design. These models are specifically designed to analyze the time from a starting point (end of treatment) until the occurrence of an event (relapse), allowing for the inclusion of both categorical and continuous covariates that explain the risk of the event (50). The analysis was performed using the phreg () function from the mets package, which calculates robust variance estimators to obtain adjusted standard errors. This modeling approach allows for the estimation of the effect of explanatory variables on the hazard of the event of interest without requiring specification of the event time distribution (51).

In the first phase of the analysis, univariate Cox regression models adjusted for the study group were constructed to identify covariates associated with relapse risk, using a significance threshold of *p* < 0.05. Subsequently, the selected covariates were included in a multivariate model, which was refined using the step () function to obtain the most parsimonious model through stepwise selection based on the Akaike Information Criterion (AIC). Hazard ratios (HRs) estimated in the final model were presented alongside their 95% confidence intervals for each covariate in the form of a forest plot. To account for baseline heterogeneity and potential confounding factors, sex, age, and MUAC were included as control covariates.

## 3 Results

**Figure 1:**
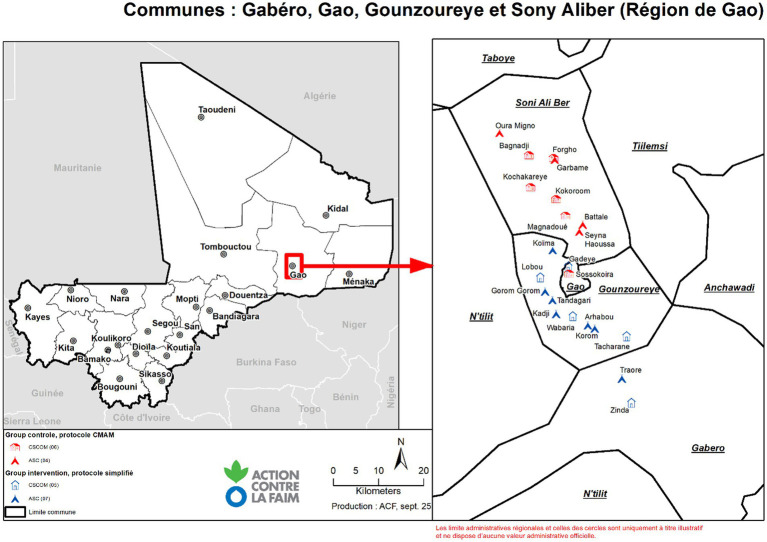
Map of the study area.

The final sample size achieved was 270 children in the simplified protocol group and 236 in the standard one, representing 93.1 and 81.3% of the initially planned sample size, respectively. Baseline characteristics at admission by study group are presented in Table 1. No significant differences at admission found between groups in terms of sex, age, or treatment provider. However, the simplified protocol group had lower MUAC values and a higher proportion of edema cases on admission. In the simplified protocol group, 94.4% (255/270) were admitted based on MUAC and 5.6% (15/270) based on edema. In the standard protocol group, 36.9% (87/236) of children were admitted based on both WHZ and MUAC, 29.7% (70/236) based on WHZ only, 17.4% (41/236) based on MUAC only, and 2.1% (5/236) based on edema. In the standard protocol group, 14.0% (33/236) of children were discharged early as cured; despite not meeting the anthropometric recovery criteria, this is considered as program implementation mistakes. No such cases were observed in the simplified protocol group. To identify these early discharges, subgroups were defined within the standard protocol group: edema, WHZ only, MUAC only, both criteria, and early discharge (Figure 1).

**Table 1:** Treatment variables of children admitted to the study compared by group.

Admission characteristics	Control *N* = 236	Simplified *N* = 270	*p*-value
Sex Female	55.5% (131)	53.3% (144)	0.624
Age (months)	15.6 ± 7.4	15.3 ± 6.6	0.762
< 12 months	37.7% (89)	35.2% (95)	0.623
12–24 months	44.9% (106)	51.5% (139)	
≥ 24 months	17.4% (41)	13.3% (36)	
Treatment provider			
Health staff	85.6% (202)	80.7% (218)	0.181
CHWs	14.4% (34)	19.3% (52)	
Anthropometry on admission			
WHZ	−3.43 ± 0.93	--	--
< − 4.0 z-score	20.3% (48)	--	--
MUAC (mm)	112.8 ± 5.6	110.6 ± 3.7	0.015
<110 mm	16.5% (39)	22.6% (61)	0.352
Oedema	3.8% (9)	5.9% (16)	0.302
Discharge characteristics			
Length of stay (days)	41.9 [35–49]	54.9 [42–70]	<0.001
RUTF sachets received	103.7 [85–120]	73.10 [49–98]	0.011
RUTF stockout	21.6% (51)	2.6% (7)	0.007
Stockout days	12.4 [7.0–15.0]	6.9 [6.0–7.0]	0.430
Anthropometry on discharge			
WHZ	−0.74 ± 0.57	--	
MUAC (mm)	124.6 ± 4.1	126.5 ± 1.7	0.173
Anthropometric gain			
Weight (g/kg/day)	6.3 ± 2.6	--	
MUAC/day (mm)	0.30 ± 0.14	0.32 ± 0.11	0.257

CHWs, Community Health Workers; MUAC, Middle Upper Arm Circumference; RUTF, Ready-to-Use Therapeutic Food; WHZ, Weight-for-Height Z-score.

**Figure 2:**
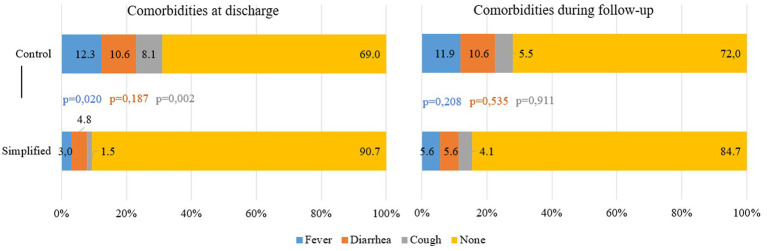
Comorbidities recorded at the moment of discharge from the treatment and during the follow-up period for relapses.

During treatment, the LoS was significantly longer in the simplified protocol group, with an average difference of nearly 2 weeks compared to the standard protocol one (54.9 vs. 41.9 days, *p* < 0.001), although no significant difference was observed in MUAC gain per day. Children in the simplified protocol group received 31 fewer RUTF sachets per cured child. The proportion of children who experienced RUTF shortages during treatment was 19% higher in the standard protocol group. Similarly, the proportion of comorbidities at discharge was 21.7% higher in the standard protocol group, with significant differences observed for fever and cough. During follow-up, this difference decreased to 12.7%, although it was no longer statistically significant (Figure 2).

Most children in both groups received two follow-up visits during the 8-month post-discharge period, Table 2. A significantly higher relapse rate was observed in the standard protocol group compared to the simplified protocol group (39.8% vs. 5.6%; Table 3). This difference was consistent across all study subgroups and follow-up months, reaching statistical significance in the fifth and sixth months. None of the 33 early-discharged children in the control group have relapsed during the study follow-up. In the standard protocol group, the highest proportion of relapses occurred in the third month (34.5%), whereas no relapses were recorded in the simplified protocol group during that period. The highest relapse rate in the simplified protocol group was 4.2%, observed during the seventh and eighth months of follow-up. A greater proportion of children relapsed to MAM in both groups. No relevant differences were found in terms of sex, treatment provider, or RUTF stock shortages during treatment. Most relapses occurred in children under 12 months of age.

**Table 2:** Follow-up visits to assess cases of relapse during the 8-month follow-up period compared by study group*.

	Control *N* = 236 % (*n*)	Simplified *N* = 270 % (*n*)	*p*-value
Follow-up visits received			
1 time	36.9% (87)	30.7% (83)	0.920
2 times	50.9% (120)	38.5% (104)	
3 times	11.4% (27)	30.0% (81)	
4 times	0.9% (2)	0.7% (2)	
Total children assessed each month after recovery			
2nd month	3.4% (8)	14.4% (39)	0.001
3rd month	24.6% (58)	21.9% (59)	0.591
4th month	43.6% (103)	36.7% (99)	0.094
5th month	16.5% (39)	25.2% (68)	0.325
6th month	17.4% (41)	20.4% (55)	0.613
7th month	39.0% (92)	44.1% (119)	0.836
8th month	14.4% (34)	17.8% (48)	0.568

*For security reasons, it was not possible to conduct monthly follow-ups. Each child received between 1 and 4 assessments done in a period between the 2nd and 8th month after recovery.

**Table 3:** Relapses recorded during the follow-up period up to 8 months after recovery.

	Control % (*n*/*N*)	Simplified % (*n*/*N*)	*p*-value	β (IC95%)
Total relapses recorded	39.8% (94/236)	5.6% (15/270)	<0.001	−2.887 [−4.946, −1.416]
By type				
Relapses to MAM	28.8% (68/236)	3.7% (10/270)	0.008	−1.822 [−5.700, 0.050]
Relapses to SAM	11.0% (26/236)	1.9% (5/270)	0.001	−2.662 [−5.701, 0.050]
By treatment provider				
Health center	41.5% (83/200)	5.5% (12/220)	0.006	−1.931 [−4.378, −0.539]
CHWs	30.6% (11/36)	6.0% (3/50)	0.007	−3.240 [−6.448, −1.053]
By sex				
Male	47.6% (50/105)	4.0% (5/126)	<0.001	−3.249 [−5.486, −1.749]
Female	33.6% (44/131)	6.9% (10/144)	0.010	−2.367 [−4.792, −0.780]
By age at discharge				
< 12 months	55.1% (49/89)	9.5% (9/95)	0.001	−3.060 [−5.589, −1.387]
12–24 months	33.0% (35/106)	4.3% (6/139)	0.010	−2.244 [−4.149, −0.681]
>24 months	24.4% (10/41)	0.0% (0/36)	1.000	--
By RUTF stock-out				
Yes	41.2% (21/51)	14.3% (1/7)	0.269	--
No	39.5% (73/185)	5.3% (14/263)	<0.001	−3.016 [−5.061, −1.540]
By comorbidities at discharge				
Yes	41.0% (16/39)	0% (0/17)	1.000	--
No	39.6% (78/197)	5.9% (15/253)	0.001	−2.840 [−4.888, −1.338]
By month (SAM+MAM)				
2nd month	25.0% (2/8)	2.6% (1/39)	0.051	--
3rd month	34.5% (20/58)	0.0% (0)	1.000	--
4th month	23.3% (24/103)	4.0% (4/99)	0.119	--
5th month	28.2% (11/39)	2.9% (2/68)	0.032	−2.704 [−6.540, −0.424]
6th month	22.0% (9/41)	1.8% (1/55)	0.020	−2.754 [−6.536, −0.473]
7th month	16.3% (15/92)	4.2% (5/119)	0.176	--
8th month	38.2% (13/34)	4.2% (2/48)	0.051	--

MAM, Moderate Acute Malnutrition; RUTF, Ready-to-Use Therapeutic Food; SAM, Severe Acute Malnutrition.

To enable comparison between matched samples, complementary analyses were conducted by excluding WHZ-only cases in the control group and retaining only children who were admitted with MUAC < 115 mm and discharged with MUAC ≥ 125 mm. This allowed for analysis of the isolated effect of the change in RUTF dosage, aligning anthropometric criteria for admission, discharge, and relapse. The sample size for the standard protocol group was reduced to 122 children (26.2% treated by CHWs), while the simplified protocol group remained unchanged (270 children, 19.3% treated by CHWs). Admission characteristics remained like those of the total sample, with no differences between groups in terms of sex, age, treatment provider, or severity at admission. However, the simplified protocol group showed higher LoS, lower RUTF consumption, and greater MUAC gain per day, while the standard protocol group had a higher proportion of RUTF stock-outs during treatment (Supplementary Table S2). Similarly, a higher proportion of children in the standard protocol group presented comorbidities at discharge and during follow-up (Supplementary Figure S1). In this MUAC-only subsample, the proportion of relapses remained higher in the standard protocol group, although statistical significance was lost (Table 4). The monthly distribution of relapses during follow-up mirrored that of the total sample, but no significant differences between groups were found after adjusting for the cluster effect.

**Table 4:** Relapses recorded during the follow-up period up to 8 months after recovery matching anthropometric criteria of the study groups*.

	Control % (*n*/*N*)	Simplified % (*n*/*N*)	*p*-value	β (IC95%)
Total relapses recorded	23.0% (28/122)	5.6% (15/270)	0.056	--
By type				
Relapses to MAM	21.3% (26/122)	3.7% (10/270)	0.031	−2.529 [−6.955, 1.841]
Relapses to SAM	1.6% (2/122)	1.9% (5/270)	0.933	--
By treatment provider				
Health center	27.3% (24/88)	5.5% (12/220)	0.056	--
CHWs	11.8% (4/34)	6.0% (3/50)	0.511	--
By sex				
Male	23.4% (11/47)	6.9% (10/144)	0.064	--
Female	22.7% (17/75)	4.0% (5/126)	0.096	--
By age at discharge				
< 12 months	35.6% (16/45)	9.5% (9/95)	0.062	--
12–24 months	20.0% (12/60)	4.3% (6/139)	0.230	--
>24 months	0.0% (0/17)	0.0% (0/36)	1.000	--
By RUTF stock-out				
Yes	21.7% (5/23)	14.3% (1/7)	0.701	--
No	23.2% (23/99)	5.3% (14/263)	0.035	−2.303 [−5.059, −0.259]
By comorbidities at discharge				
Yes	29.2% (7/24)	0% (0/17)	1.000	--
No	21.4% (21/98)	5.9% (15/253)	0.005	−2.708 [−5.117, −0.945]
By month (SAM+MAM)				
2nd month	16.7% (1/6)	2.6% (1/39)	0.303	--
3rd month	25.7% (9/35)	0.0% (0)	1.000	--
4th month	12.7% (7/55)	4.0% (4/99)	0.540	--
5th month	22.7% (5/22)	2.9% (2/68)	0.211	--
6th month	4.5% (1/22)	1.8% (1/55)	0.842	--
7th month	9.5% (4/42)	4.2% (5/119)	0.352	--
8th month	11.1% (2/18)	4.2% (2/48)	0.303	--

MAM, Moderate Acute Malnutrition; RUTF, Ready-to-Use Therapeutic Food; SAM, Severe Acute Malnutrition. *Selecting children in the control group admitted and discharged by MUAC and excluding those admitted by WHZ only. The simplified group stays the same.

**Figure 3:**
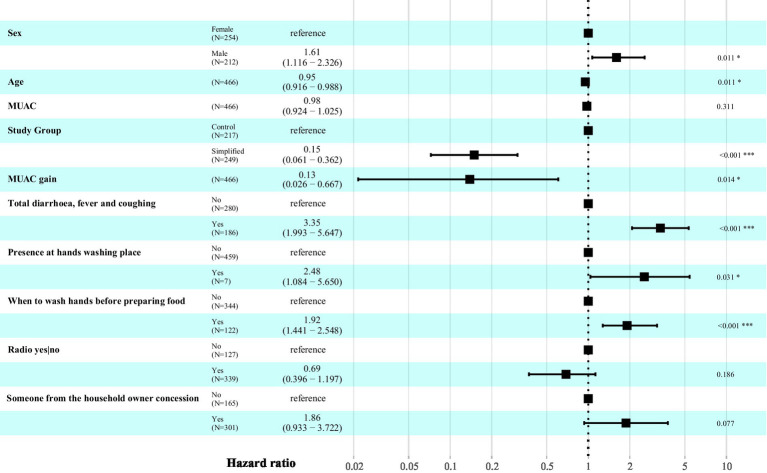
Variables associated with relapse risk using a Cox proportional hazards model.

To identify the profile of children at the highest risk of relapse and factors associated, the influence of 106 variables was analyzed (Supplementary Table S1). The multivariate Cox model, adjusted on the overall sample, identified seven variables significantly associated with relapse risk (Figure 3). Protective factors—those that reduced the risk of relapse over time—included: being treated in the simplified protocol group versus the standard protocol group, which reduced the risk by 85% (HR: 0.15; 95% CI: 0.06–0.36); each additional month of age at discharge, which reduced the risk by 5% (HR: 0.95; 95% CI: 0.92–0.99); and each additional millimeter of MUAC gain, which reduced the risk by 87% (HR: 0.13; 95% CI: 0.03–0.67). Conversely, the main risk factors for relapse were the presence of comorbidities during follow-up, which tripled the risk (HR: 3.35; 95% CI: 1.99–5.65); the presence of a handwashing point (HR: 2.48; 95% CI: 1.08–5.65); handwashing before food preparation (HR: 1.92; 95% CI: 1.44–2.55); and male sex of the children (HR: 1.61; 95% CI: 1.12–2.33). These hazard ratios (HRs) reflect the individual effect of each variable, adjusted for the others in the model. Therefore, the combined effect must be calculated separately.

**Figure 4:**
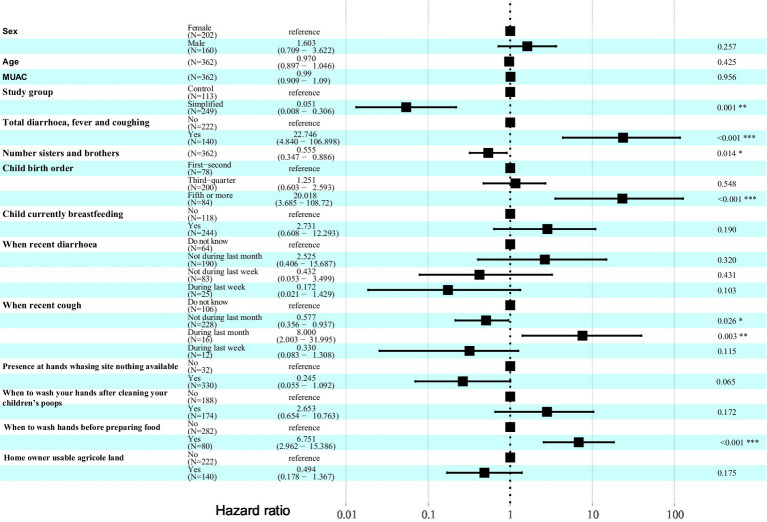
Variables associated with relapse risk using a Cox proportional hazards model for the MUAC-only subsample.

The Cox model adjusted for the MUAC-only subsample (i.e., matching anthropometric criteria for admission, discharge, and relapse) revealed a more complex picture of relapse risk, with a greater number of variables involved (Figure 4). Protective factors included: treatment with the simplified protocol, which reduced relapse risk by 95% (HR: 0.051; 95% CI: 0.008–0.31); number of siblings, with each additional sibling reducing risk by 45% (HR: 0.55; 95% CI: 0.35–0.89); absence of cough in the last month of follow-up (HR: 0.57; 95% CI: 0.36–0.94). Risk factors in this model included: presence of any comorbidity during follow-up, which increased relapse risk nearly 20-fold (HR: 22.75; 95% CI: 4.84–106.90); being the fifth child or higher in birth order (HR: 20.02; 95% CI: 3.68–108.72); presence of cough in the last month of follow-up (HR: 8.00; 95% CI: 2.00–31.99); and handwashing before food preparation, which increased the risk nearly sevenfold (HR: 6.75; 95% CI: 2.96–15.39).

## 4 Discussion

The results of this study confirm the risk of relapses among children previously treated for SAM. In the Gao region, 39.8% of children treated with the standard protocol and 5.6% of those treated in the simplified protocol experienced a new episode of acute malnutrition during the 8-month post-discharge follow-up. This difference persisted across both HFs and CHW settings. These results were also confirmed when we analyzed only the sub-sample of children admitted using only the MUAC criterion within the control group. The relapse rate in our standard control group was comparable with previous studies but was apparently lower in the simplified protocol group.

Relapse rates vary widely depending on the context, the definition of relapses, and the follow-up period. Reported relapse rates range from less than 1% in Bangladesh O’Sullivan et al. (41), to 15.2% in Ethiopia Girma et al. (52), 19% in Somalia and up to 63% in South Sudan King et al. (53). The proportion of relapses we found in the standard protocol is high, similar to most of the studies published, and also similar to other region in Mali, Kayes (not an emergency setting) when it found a 30% of relapses in children suffering from acute malnutrition (53). Similarity, Brander et al. (54) in a recent study evidenced that 49.4% in Mali and 61.5% of children in Burkina Faso experienced a relapse during the 6 month follow up.

In our study, the relapse rate to SAM was significantly lower in the simplified protocol group (1.9% vs. 11%) and it was not reported before with this protocol. Lelijveld et al. (43) in Kenya implemented the same ComPAS protocol, found no significant difference in relapses to SAM between groups, with a rate of 3% in both (*p* = 0.72) at 4 months post-discharge, and relapse to MAM of 10% in the simplified group versus 12% in the standard one (*p* = 0.34). Kangas et al. (45) in Mali, in a cohort of children treated under the same ComPAS protocol (without a control group), reported a relapse rate to SAM of 1.7% (95% CI: 0.6–3.6), and to MAM of 26.1% (95% CI: 21.7–30.8) at 6 months of follow-up. As in previous studies, we observed a higher proportion of children relapsing to MAM, but significantly lower in the simplified protocol group (3.7% vs. 28.8%) and lower than the figures reported in the studies with this same protocol.

Analyzing other modified protocols, Kangas et al. (55) in Burkina Faso, applying the MANGO protocol (Modeling an Alternative Nutrition Protocol Generalizable for Outpatient care), which uses a standard RUTF dose for 2 weeks followed by a reduced dose, found no significant difference in relapse to SAM compared to the standard protocol (2.4% in the control group vs. 1.8% in the intervention group, *p* = 0.69). Ntaongo-Alendi et al. (56) in the Democratic Republic of Congo, using the same MANGO protocol, reported relapse rates of 2.4% in the standard group and 2.9% in the intervention group (*p* = 0.75) using the MANGO protocol over a three-month follow-up period. Daures et al. (44) in Burkina Faso, using the OPTIMA protocol without control group which propose the reducing of RUTF dose by increasing the weight or MUAC rather than increasing the RUTF (children with MUAC< 115 mm or edema receive 175 kcal/kg/day, those with MUAC between 115 and 119 mm receive 125 kcal/kg/day, and children with MUAC> 120 mm receive 75 kcal/kg/day), reported relapse rates of 3.7% to SAM and 2.4% to MAM within 3 months post-discharge. This low rate may be partly explained by the distribution of RUSF (ready-to-use supplementary food) by the World Food Program as a preventive measure but is consistent with our study. Daures et al. (57) in 3-arm individually randomized, controlled, noninferiority trial which compared the standard protocol with ComPAS and OPTIMA in Niger, found no difference in relapses between groups at 6 months follow up.

Regarding risk factors for relapses, several studies studied anthropometric measurements on admission. In our study, the group applying the simplified protocol with lower relapses rates, had a lower MUAC 110.6 vs. 112.8. This is not confirmed by other studies. Adegoke et al. (58) in Nigeria (24% relapse to SAM), Kangas et al. (45) in Mali (1.7%), and Daures et al. (44) in Burkina Faso (3.7%) reported the association of lower anthropometry at admission with higher risk of relapses. Early identification of malnutrition cases, through strategies such as family MUAC, can serve as a preventive measure to reduce relapse rates at the community level.

Low anthropometric measurements at discharge are also associated with increased relapse risk. In our study, we observed difference in MUAC at discharged, lower in the control one which had a higher proportion of relapses, 124.6 vs. 126.5 standard protocol and simplified protocol group respectively, even if it was not statistically significant. These funding are consistent with Sommasé et al. (59), Blizshanska et al. in Niger (60), King et al. (53) in Mali, Somalia, and South Sudan, Kangas et al. (45) in Mali, and Lelijveld et al. (43) in Kenya; and also, with Stobaugh et al. (42), in her systematic review. Continuing analyzing anthropometry at discharge, 14% of children in the standard protocol group in our study were discharged as cured without meeting the anthropometric recovery criteria, compared to none in the simplified protocol group. This could potentially contribute to the lower MUAC at discharged in the standard protocol group. These cases represent errors in case management, and it was previously reported under standard protocols involving WHZ in Mali, López-Ejeda et al. (18) and Niger Charle-Cuéllar et al. (15). This finding supports earlier evidence from a systematic review by Stobaugh et al. (42), as well as studies in Nepal, Guesdon et al. (61), and Ethiopia, Lambebo et al. (62), which found that children under five discharged without meeting WHO recovery criteria had a higher risk of relapse.

Few studies examined average LoS as a potential factor influencing relapses, and findings remain inconclusive. Our study found that children treated under the simplified protocol, who had a longer LoS than those in the standard protocol (54.9 vs. 41.9 days), had a significantly lower relapse rate. A similar result reported by Hitchings et al. (63) in Nigeria, where children with longer treatment durations had a lower risk of relapse within 3 months post-discharge. In contrast, Daures et al. (44) in Burkina Faso reported that children with a mean LoS > 5 weeks had higher relapse rates. This longer treatment time does not appear to be related to lower weight gain, presumably due to a lower amount of RUTF, since the MUAC at discharge is higher in the group that used a simplified protocol.

In our study, comorbidities during follow-up emerged as the most robust predictor of relapse, associated with an estimated 20-fold increase in risk. A higher proportion of children suffered from fever and diarrhea in the standard protocol group. Regarding morbidity, there is no single profile that defines the risk of relapse. Some studies show an increased risk of relapse when conditions such as fever or diarrhea occur during the six-month follow-up period. Stobaugh et al. (42), in her systematic review, identified fever as a factor associated with relapses after discharge. Similarly, King et al. (54) in Mali and South Sudan found that relapses were associated with diarrhea or fever during follow-up. Kangas et al. (45), in a longitudinal cohort study in Mali using the ComPAS protocol, also identified morbidity during follow-up as a risk factor for relapse. Adegoke et al. (58) reported that an episode of diarrhea during the first month of follow-up was associated with relapse in Nigeria.

The presence of morbidity at discharge may support the hypothesis that anthropometric recovery does not necessarily coincide with immunological recovery in children treated for malnutrition. This could explain the higher relapse rates observed in our standard protocol group. Blizshanska et al. (56) explored this hypothesis, suggesting that the high incidence of morbidity during the post-discharge period may be due to anthropometric recovery outpacing immunological restoration. Chevalier et al. (64) in Bolivia showed that children treated for SAM recovered WHZ after 2 weeks, MUAC after 4–5 weeks, while immunological recovery occurred only after the eighth week of treatment. Blizshanska et al. (65) in a recent meta-analysis about optimal anthropometric discharge criteria from treatment of wasting, suggest that children are at increased risk of morbidity post-discharge despite meeting anthropometric criteria. These data are striking and support the hypothesis that immunological recovery may lag behind anthropometric recovery and be incomplete at the time of programmatic discharge. Brander et al. (54), evidenced the effect of small-quantity lipid-based nutrient supplements (SQ-LNS) in preventing relapses. This is plausible because SQ LNS provide micronutrients that are important for immunity, particularly zinc, and can help restore immunity, which does not seem to have been achieved during anthropometric recovery. Taking together these findings could support the hypothesis that the longer LoS observed in the simplified protocol group, combined with the absence of early discharges, may contribute to improved immune recovery, fewer comorbidities during follow-up, and ultimately a lower relapse rate.

In identifying the socio-economic characteristics associated with an increased risk of relapse, findings across studies appear heterogeneous. The influence of these variables seems to vary by country, highlighting the need to tailor mitigation strategies to local contexts. Moreover, no consistent association was found between socio-economic variables and relapse risk based on the type of treatment protocol received, whether standard or simplified. In our study, we found no association between household food insecurity and relapse. King et al. (53) identified food insecurity, driven by the prolonged humanitarian crisis in South Sudan, as a risk factor for relapse. In contrast, Somalia, the country which observed the lowest relapse rates, had a higher proportion of families participating in food assistance programs, which might have a protective effect. Similarly, Abitew et al. (66) in Ethiopia and Adegoke et al. (58) in Nigeria found that children from food-insecure households had a higher risk of relapse after treatment. However, other studies did not confirm this association. Lelijveld et al. (43) in Kenya found no relationship between food insecurity and relapse, and Stobaugh et al. (67) in Malawi reported no effect of nutritional supplementation on relapse rates among children treated for MAM, although the population differed from those treated for SAM.

Regarding water, sanitation, and hygiene (WASH) conditions, the lack of harmonization in variables collected across studies makes comparisons difficult. Contrary to expectations, the presence of a handwashing station at the household level and the practice of washing hands before food preparation was associated with a higher risk of relapse. These associations should not be interpreted as direct causal relationships, but rather as context-specific findings and suggest that good hygiene practices or infrastructure do not necessarily guarantee the quality of water used for hygiene purposes. D’Mello-Guyett et al. (68) in Mali evidenced that the lack of soap at the hand washing and the use of multiple water sources are associated with relapses. Kangas et al. (45) identified improved water use as a protective factor against relapses. Abitew et al. (66) reported similar findings, linking relapses to limited access to improved water sources (defined as living more than 15 min away) and poor hand hygiene practices. A possible causal relationship between WASH conditions and acute malnutrition remains debated. McLeod et al. (69), in a systematic review including three studies on this topic, found inconsistent associations with relapse. Notably, Altmast et al. (70) in Chad evaluated the effect of a WASH package for children treated for SAM and found no difference in relapse rates between groups at two- and six-months post-discharge. Although results may appear contradictory, the hypothesis that WASH conditions influence relapse risk remains plausible and warrants further investigation.

The present study has several limitations. First, it was not designed as a randomized controlled trial, which inherently limits the strength of causal inferences. Therefore, the results should be interpreted with caution and are not generalizable to other settings or populations without further validation. Second, because of the low sample size, some risk factors may have remained undetected. Additionally, during the follow-up children admitted in the study could not be monitored monthly, and the number and timing of follow-up visits varied considerably each month after recovery. This variability affected both the simplified and standard protocol groups. These limitations are largely attributable to the operational constraints of working in an unstable and insecure environment, which posed significant challenges to the implementation and monitoring of the study. Despite these constraints, the findings provide valuable insights and highlight areas for further investigation under more controlled conditions. Finally, the studies referenced in our discussion did not follow a standardized methodology for defining relapses; some used different anthropometric criteria and follow-up periods after discharge. The Council of Research & Technical Advice for Acute Malnutrition (CORTASAM) proposed a theoretical framework to standardize relapse studies Schaefer et al. (71). This publication harmonized the definition of relapses, follow-up procedures, and potential influencing factors. Since then, few studies on relapse have been published, and only King et al. (53) has followed this methodology.

## 5 Conclusion

This study reinforces the growing body of evidence that relapses after treatment for SAM remains a public health challenge, particularly in emergency contexts. Our findings suggest that the use of a simplified protocol may lead to fewer errors when applying the protocol and to lower relapse rates, even when reduced quantities of RUTF are administered. Comorbidities during follow-up emerged as the most consistent predictor of relapses across settings. These results underscore the importance of improving discharge procedures, ensuring adequate post-discharge monitoring, and integrating context-specific strategies to sustain recovery. The integration of malnutrition management with other health services should be considered by policymakers to monitor children recovering from SAM who remain at risk. Growth monitoring offers an opportunity to track child development and enable early identification of stagnation or associated pathologies. More robust and standardized studies, aligned with the CORTASAM framework and incorporating various protocol simplifications, as well as studies incorporating SQ-LNS as a post-discharge action after treatment are urgently needed to inform policy and improve long-term outcomes for children recovering from acute malnutrition.

## Data Availability

The raw data supporting the conclusions of this article will be made available by the authors, without undue reservation.
