# Supplementary material for: Reducing relapses after acute malnutrition treatment: evidence from a simplified approach in emergency settings of Mali
Source: Front Public Health. 2026 Mar 12;14:1773585. doi: 10.3389/fpubh.2026.1773585 (PMC13020562; doi:10.3389/fpubh.2026.1773585)

Reducing relapses after acute malnutrition treatment: evidence from a simplified approach in emergency settings of Mali

Supplementary Material

| **Table S1.** Variables included in the Cox regression models to assess impact on the relapse risk. | | |
| --- | --- | --- |
| **Group** | **Total number** | **Variables** |
| **Anthropometric** | **4** | Age at discharge |
|  |  | MUAC at admission |
|  |  | MUAC at discharge |
|  |  | MUAC gain |
| **Demographics** | **8** | Sex |
|  |  | Sex of caregiver |
|  |  | Relation of caregiver and child |
|  |  | Father alive |
|  |  | Child twin |
|  |  | Number of sisters and brothers |
|  |  | Child birth order |
|  |  | Number of household inhabitants |
| **Treatment** | **6** | Protocol of treatment (CMAM standard or Simplified) |
|  |  | Treatment provider (HF or CHW) |
|  |  | Time to recovery (days) |
|  |  | Early discharge from treatment |
|  |  | RUTF sachets received |
|  |  | RUTF stock-out duration (days) |
| **Comorbidities** | **4** | When recent diarrhea at discharge |
|  |  | When recent fever at discharge |
|  |  | When recent cough at discharge |
|  |  | Diarrhea, fever or cough during follow-up period |
| **Food security and feeding practices** | **7** | Child currently breastfeed |
|  |  | Age child first food intake (months) |
|  |  | Age child first liquids intake (months) |
|  |  | Child was ever breastfed |
|  |  | Last four weeks, complete lack of food in the household |
|  |  | Last four weeks, anyone in the household sleeps without eating? |
|  |  | Last four weeks, any full day without eating? |
| **Healthcare access** | **4** | Distance from household to the treatment provision site |
|  |  | Mean of transportation used |
|  |  | Transportation time spent |
|  |  | Child never vaccinated |
| **Water, sanitation and hygiene** | **47** | Origin of drinking water: surface |
|  |  | Origin of drinking water: household tap |
|  |  | Origin of drinking water: private tap on plot |
|  |  | Origin of drinking water: neighbor's tap |
|  |  | Origin of drinking water: communitary tap |
|  |  | Origin of drinking water: well pump drilling |
|  |  | Origin of drinking water: protected open dug well |
|  |  | Origin of drinking water: unprotected open dug well |
|  |  | Origin of drinking water: protected source |
|  |  | Origin of drinking water: non protected source |
|  |  | Origin of drinking water: rainfall |
|  |  | Origin of drinking water: tanker |
|  |  | Origin of drinking water: tank cart |
|  |  | Origin of drinking water: kiosk |
|  |  | Origin of drinking water: bottle |
|  |  | Origin of drinking water: sacket |
|  |  | Origin of drinking water: other |
|  |  | Place of drinking water |
|  |  | How long to go and return to the water source? |
|  |  | Other alternative sources of drinking water used |
|  |  | Last four weeks, frequency of insufficient_drinking_water |
|  |  | Method for purifying water |
|  |  | Type of usual household latrine |
|  |  | Location of latrines |
|  |  | Public shared toilets |
|  |  | Where does the child defecate? |
|  |  | Last type of defecation of the child |
|  |  | Place for hand washing |
|  |  | Where do household members wash their hands? |
|  |  | Water available to hand washing |
|  |  | Presence at hand washing site: soap |
|  |  | Presence at hand washing site: laundry |
|  |  | Presence at hand washing site: ash, mud or sand |
|  |  | Presence at hand washing site: nothing |
|  |  | Handwashing moment: after defecation |
|  |  | Handwashing moment: after cleaning children's poops |
|  |  | Handwashing moment: after changing bed for children |
|  |  | Handwashing moment: before preparing food |
|  |  | Handwashing moment: before eating food |
|  |  | Handwashing moment: before feeding children |
|  |  | Handwashing moment: after eating food |
|  |  | Handwashing moment: returning from outside work |
|  |  | What do you use to wash hands?: only water |
|  |  | What do you use to wash hands?: water and soap |
|  |  | What do you use to wash hands?: water and sand |
|  |  | What do you use to wash hands?: water and ash |
|  |  | What do you use to wash hands?: other |
| **Socioeconomic level and livelihoods** | **26** | Household commodities: mattress |
|  |  | Household commodities: mobile phone |
|  |  | Household commodities: fridge |
|  |  | Household commodities: television |
|  |  | Household commodities: radio |
|  |  | Household commodities: table |
|  |  | Household commodities: bench chair |
|  |  | Household commodities: torch |
|  |  | Household commodities: bycicle |
|  |  | Household commodities: car, motorbike |
|  |  | Household commodities: animal-drawn cart |
|  |  | Household commodities: shoes |
|  |  | Number of rooms in the household |
|  |  | Separate kitchen in the household |
|  |  | Savings in the bank account |
|  |  | Access to electricity at household |
|  |  | Someone of the household owner concession |
|  |  | Home owner usable agricole land |
|  |  | How many hectares per household member? |
|  |  | Someone from the household who owns livestock |
|  |  | Animals ownership |
|  |  | Participation in any food support program? |
|  |  | Participation in any economical support program? |
|  |  | Participation in any WASH support program? |
|  |  | Participation in any agricultural support program? |
|  |  | Participation in any other support program? |
| CHW: Community Health Worker; CMAM: Community Management of Acute Malnutrition; HF: Health Facility; MUAC: Middle-Upper Arm Circumference; RUTF: Ready-to-Use Therapeutic Food; WASH: Water, Sanitation and Hygiene. | | |

| **Table S2.** Treatment variables of children admitted to the study compared by group matching anthropometric criteria of the study groups* | | | |
| --- | --- | --- | --- |
| **ADMISSION CHARACTERISTICS** | **CONTROL**  N=122 | **SIMPLIFIED**  N=270 | **p-value** |
| **Sex Female** | 61.5% (75) | 53.3% (144) | 0.133 |
| **Age (months)** | 15.2 ± 7.1 | 15.3 ± 6.6 | 0.940 |
| < 12 months | 36.9% (45) | 35.2% (95) | 0.922 |
| 12-24 months | 49.2% (60) | 51.5% (139) |  |
| ≥ 24 months | 13.9% (17) | 13.3% (36) |  |
| **Treatment provider** |  |  |  |
| Health staff | 73.8% (90) | 80.7% (218) | 0.154 |
| CHWs | 26.2% (32) | 19.3% (52) |  |
| **Anthropometry on admission** |  |  |  |
| MUAC (mm) | 112.4 ± 3.5 | 110.6 ± 3.7 | 0.051 |
| <110 mm | 13.9% (17) | 22.6% (61) | 0.321 |
| **Oedema** | 0.0% (0) | 5.9% (1) | 0.994 |
| **DICHARGE CHARACTERISTICS** | **CONTROL**  N=122 | **SIMPLIFIED**  N=270 | **p-value** |
| **Length of stay (days)** | 40.8 [35.0 – 49.0] | 54.9 [42.0 – 70.0] | 0.001 |
| **RUTF sachets received** | 100.2 [80.3 – 110.0] | 73.10 [49.0 – 98.0] | 0.008 |
| **RUTF stockout** | 18.9% (23) | 2.6% (7) | 0.006 |
| Stockout days | 8.0 [7.0 – 7.0] | 6.9 [6.0 – 7.0] | 0.320 |
| **Anthropometry on discharge** |  |  |  |
| MUAC (mm) | 126.8 ± 2.1 | 126.5 ± 1.7 | 0.253 |
| **Anthropometric** **gain** |  |  |  |
| MUAC/day (mm) | 0.30 ± 0.14 | 0.32 ± 0.11 | 0.010 |
| CHWs: Community Health Workers; MUAC: Middle Upper Arm Circumference; RUTF: Ready-to-Use Therapeutic Food; WHZ: Weight-for-Height Z-score. *Selecting children in the control group admitted and discharged by MUAC and excluding those admitted by WHZ only. The simplified group stays the same. | | | |

| **Table S3.** Follow-up visits to assess cases of relapse during the 8-month follow-up period compared by study group matching anthropometric criteria of the study groups*. | | | |
| --- | --- | --- | --- |
|  | **CONTROL**  N=122 **% (n)** | **SIMPLIFIED**  N=270  **% (n)** | **p-value** |
| **Follow-up visits received** |  |  |  |
| 1 time | 36.9% (45) | 30.7% (83) | 0.685 |
| 2 times | 46.7% (57) | 38.5% (104) |  |
| 3 times | 14.8% (18) | 30.0% (81) |  |
| 4 times | 1.6% (2) | 0.7% (2) |  |
| **Total children assessed**  **each month after recovery** |  |  |  |
| 2^nd^ month | 4.9% (6) | 14.4% (39) | 0.021 |
| 3^rd^ month | 28.7% (35) | 21.9% (59) | 0.502 |
| 4^th^ month | 45.1% (55) | 36.7% (99) | 0.081 |
| 5^th^ month | 18.0% (22) | 25.2% (68) | 0.622 |
| 6^th^ month | 18.0% (22) | 20.4% (55) | 0.708 |
| 7^th^ month | 34.4% (42) | 44.1% (119) | 0.848 |
| 8^th^ month | 14.8% (18) | 17.8% (48) | 0.568 |
| *Selecting children in the control group admitted and discharged by MUAC and excluding those admitted by WHZ only. The simplified group stays the same. For security reasons, it was not possible to conduct monthly follow-ups. Each child received between 1 and 4 assessments done in a period between the 2nd and 8th month after recovery. | | | |

**Figure S1.** Comorbidities recorded at discharge from the treatment and during the follow-up period for relapses compared by group matching anthropometric criteria*

*Selecting children in the control group admitted and discharged by MUAC and excluding those admitted by WHZ only. The simplified group stays the same


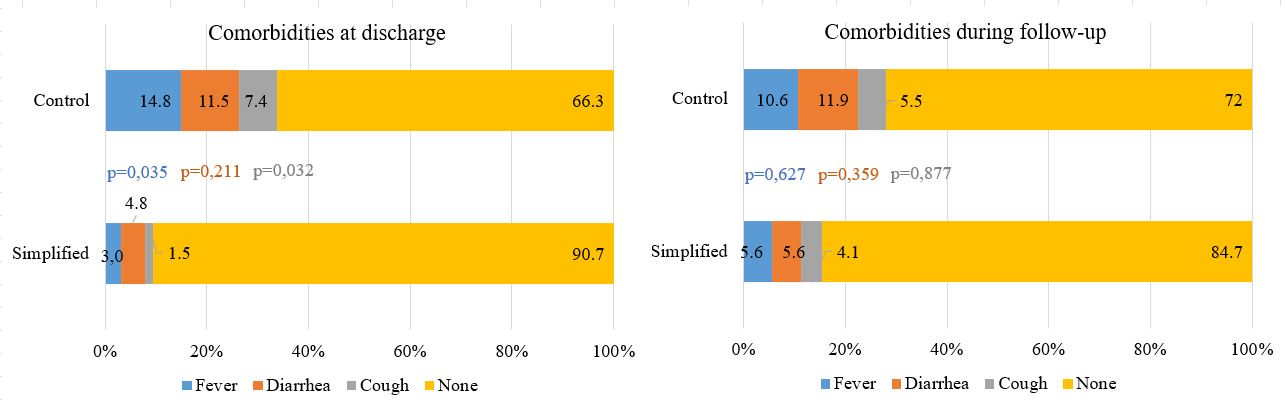

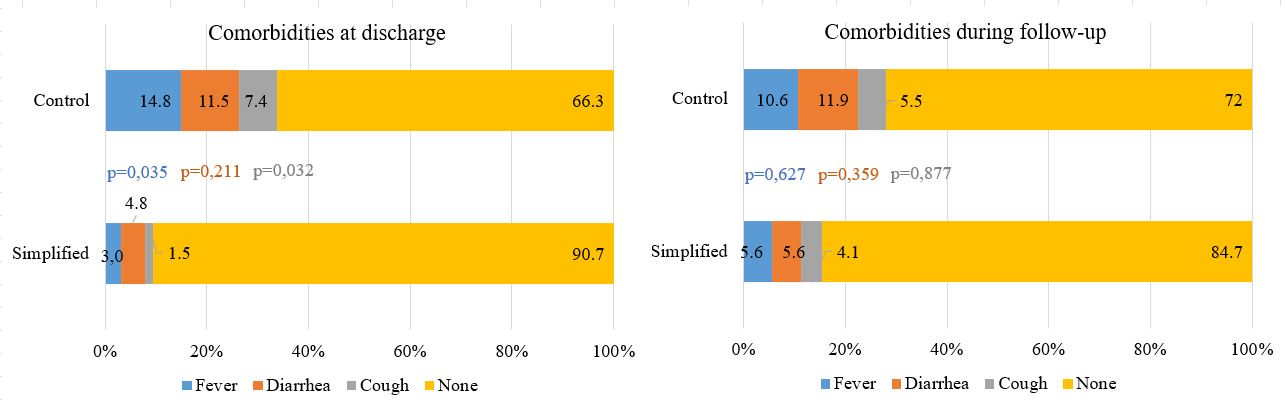

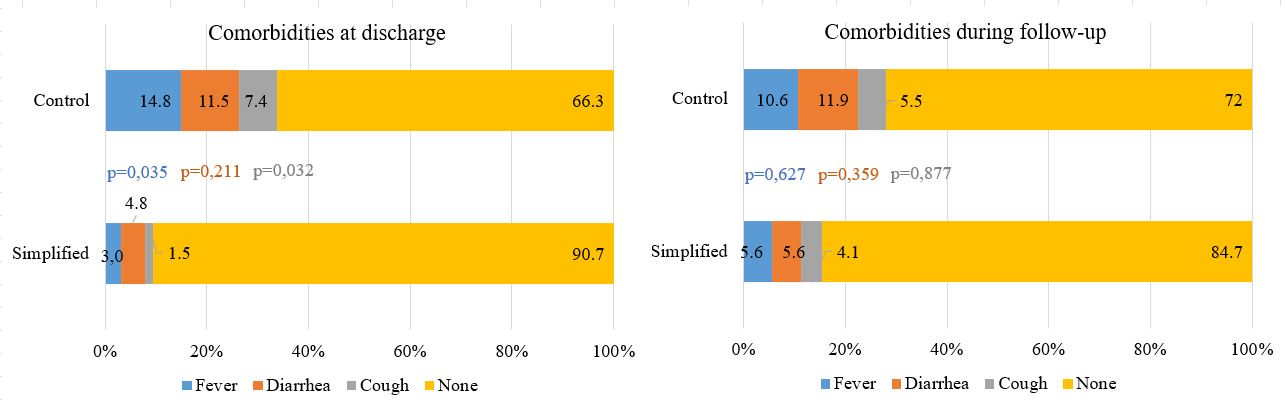

Supplement: Supplementary file 1 [file Supplementary_file_1.docx]
